# Tremulous spastic ataxia in a patient with a homozygous truncating *SYNE1* variant

**DOI:** 10.1016/j.prdoa.2023.100205

**Published:** 2023-06-07

**Authors:** Francesca Spagnolo, Edoardo Monfrini, Vincenza Pinto, Giovanni Di Maggio, Paolo De Marco, Giacomo P. Comi, Augusto Rini, Alessio Di Fonzo

**Affiliations:** a Neurological Department, A. Perrino's Hospital, Brindisi, Italy; b Fondazione IRCCS Ca’ Granda Ospedale Maggiore Policlinico, Neurology Unit, Milan, Italy

**Keywords:** SYNE1, ARCA, Ataxia, Spasticity, Tremor

## Abstract

We describe a case of severe adult-onset progressive tremulous cerebellar ataxia with pyramidal signs associated with a rare homozygous truncating pathogenic variant in the *SYNE1* gene (p.Arg5371*). This contrasts the initial views on SYNE1-related ataxia as a relatively benign, slowly progressive condition, with important implications for clinic-genetic counselling.

*SYNE1* is one of the largest genes in the human genome, encoding a protein of the spectrin family, Nesprin1, which forms an intra-cellular network linking organelles with the actin cytoskeleton [1]. It is highly expressed in skeletal muscle, heart, and cerebellum, with an important function in muscle and nervous system development [1].

In humans, pathogenic *SYNE1* variants have been initially reported to cause a slowly progressive, relatively pure cerebellar ataxia (spinocerebellar ataxia, autosomal-recessive 8; SCAR8) [2], mainly observed in Quebec, Canada [1], [3]. SYNE1-related ataxia, inherited as an autosomal recessive disorder, typically presents with adult-onset ataxia and dysarthria, with slow progression of the disease, causing moderate levels of disability, but no impact on life expectancy. Executive functions are also generally involved. Until a few years ago *SYNE1* mutations were thought to be confined to the Canadian region [1].

In 2016, Synokzik et al. and Mademan et al., described a cohort of non-Canadian patients with SYNE1-related ataxia, indicating that *SYNE1* pathogenic variants are much more common than previously thought [4], [5]. Moreover, most patients had extracerebellar neurological signs, suggesting that the clinical phenotype is more complex than pure cerebellar ataxia. Unfortunately, multisystemic involvement seems to be the rule rather than the exception in SYNE1-related disease, and variable additional features can be detected in 81% of patients, including upper and lower motor neuron disease, brainstem dysfunction, and a variable range of musculoskeletal abnormalities [2]. Hence, this disease represents a continuum of severity phenotypes [2], [4], [5].

However, several aspects of phenotypic expression are still obscure, and more effort is required to disentangle clinical variability. In this report, we describe an Italian patient with adult-onset tremor, cerebellar ataxia, and pyramidal signs associated with a rare homozygous truncating mutation in the *SYNE1* gene.

## 1 Case report

A 26-years-old man came to our attention in 2019, complaining of bilateral tremor, particularly distressing during his gym workouts. He was the only child of non-consanguineous parents, coming from a small town in Southern Italy. The patient was born at term after an uneventful pregnancy. There was no neurological disease in his family.

The neurological examination revealed a combined tremor syndrome [6], with slight bilateral symmetrical kinetic, postural and isometric tremor in his upper limbs. A cerebellar dysarthria, associated with pyramidal signs (brisk tendon reflexes, especially affecting lower limbs, increased tone and an incorrect plantar response) were also detected. Mild upper and lower limb incoordination was present as well. Walking was autonomous and ataxic, with paretic notes; tandem gait was pathological (Video1).

Extensive investigations (including plasma and urine amino acids, urine metabolic screens, thyroid and liver function, full blood count, very long-chain fatty acids, transferrin isoforms, vitamin E levels, and alpha-fetoprotein) failed to identify an etiology. Only a slight but constant increase in creatine kinase (CK) levels could be detected.

Nerve conduction studies were normal. Brain and spinal MRI was performed and displayed marked cerebellar atrophy with preserved brainstem, spinal and supratentorial structures (FigureAFig. 1). The cardiological and ophthalmological examinations resulted normal. A formal psychometric assessment disclosed a mild dysexecutive syndrome.

**Fig. 1:**
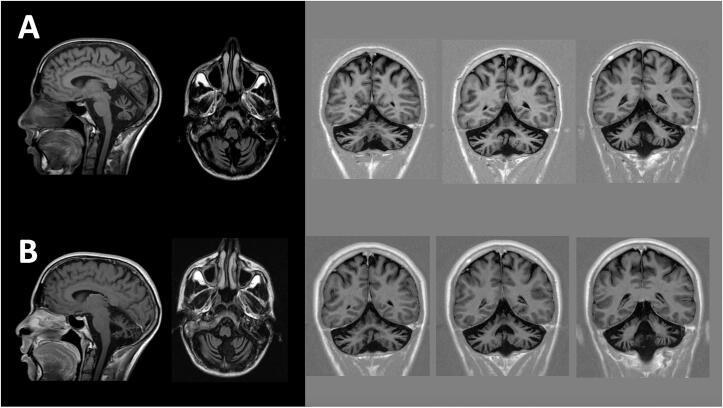
Brain MRI (T1 and T1 inversion-recovery weighted sequences) of the patient was available at baseline (A) and three years later (B), showing marked cerebellar atrophy with preserved brainstem, spinal and supratentorial structures. A slight progression of atrophy can be appreciated in the radiological follow-up, with progression in IV ventricle enlargement. **The subject gave consent to be videoed for publication both in print and online.**

Three years after the first evaluation the patient’s neurological deficits slightly progressed: his ataxic gait worsened and a pronounced slurred speech with articulation and phonological difficulties was observed (Video2). Ataxia-associated scales worsened over time, progressing from 5 to 10 points for the SARA scale [7] and from 12 to 23 points for the ICARS scale [8]. A slight progression of cerebellar atrophy is noticeable on brain MRI, with enlargement of the 4th ventricle (FigureB).

Genetic testing utilized a two-step approach: firstly, the most frequent genes responsible for hereditary ataxia were examined, including trinucleotide repeat expansions causing Friedreich’s ataxia (FRDA) and Spinocerebellar ataxias (SCA 1–2-3–6-7–17); then a second-generation sequencing approach was adopted, looking for mutations in the genes associated with cerebellar ataxia. A rare homozygous nonsense variant (NM_182961.4, c.16111C > T, p.Arg5371*) in the *SYNE1* gene was identified and confirmed by Sanger sequencing. This mutation creates a premature translational stop codon, resulting in an absent or disrupted protein product. Loss-of-function variants in *SYNE1* are known to be pathogenic. This variant has never been described in the literature; however, it is present at an extremely low frequency in population databases, only in heterozygous state (rs772587027, gnomAD Allele Frequency = 0.00001593) and reported pathogenic by a single submission in ClinVar (RCV001385856). The variant was classified pathogenic applying the following ACMG classification criteria: PVS1, PP5 and PM2. The parents of the patient were not available for sampling; however, it is reasonable to suspect a possible distant consanguinity, considering their common origin from the same small village of Southern Italy. The Ethics Committee of the A. Perrino’s Hospital (Brindisi, Italy) approved the study. Written informed consent was obtained from the patient.

## 2 Discussion

Autosomal recessive cerebellar ataxias (ARCA) represent a continuously expanding group of hereditary neurodegenerative disorders. Among them, SYNE1-related disease has been traditionally associated with adult-onset pure cerebellar ataxia. However, there is emerging evidence of clinical phenotypic diversity associated with SYNE1 mutations [3]. In up to 50% of patients, SYNE1-related disease starts with non-cerebellar features, often a combination of upper and lower motor neuron dysfunction. Synofzik et al. [4] screened 434 European ataxia patients, demonstrating a frequency of *SYNE1* mutation higher than previously thought (5.3% after exclusion of Friedreich’s ataxia and the most common repeat expansion SCAs), and that it commonly presents with a multisystemic neurodegenerative disease. This includes motor neuron and brainstem features and even complex neuromuscular syndromes, where respiratory dysfunction can lead to premature death. Mademan et al, [5] further confirmed these data, showing especially how amyotrophic lateral sclerosis (ALS)-like motor neuron involvement represents a frequent feature of biallelic SYNE1 mutations carriers (∼63% of patients). When performed, muscle biopsy showed neurogenic changes in these patients, thus corresponding to the clinical/EMG finding of frequent lower motor damage [4]. In our patient nerve conduction studies were normal, however, CK levels were constantly increased, as already described in patients carrying *SYNE1* mutations [4]. His neuropsychological profile was in line with previous reports, showing an impairment of executive functions. These findings further confirm that executive dysfunction, especially working memory, is a prevalent neuropsychological abnormality in hereditary ataxias as part of the cerebellar syndrome.

The mutational spectrum of SYNE1-related disease spreads across the gigantic *SYNE1* gene. Almost all mutations are private, indicating the need to sequence all the 146 exons. Loss-of-function of Nesprin1 is the most likely underlying mechanism in SYNE1-related disease [2], [3]. No obvious genotype-phenotype correlations could be established so far [5]. Different *SYNE1* mutations might differentially affect the multiple transcript isoforms which vary greatly in tissue-specific expression patterns.

Here we reported a case carrying a homozygous *SYNE1* nonsense pathogenic variant that further confirms and expands the clinical complexity associated with *SYNE1* mutations. Our patient’s clinical phenotype was not purely cerebellar, showing action tremor as the initial predominant sign and a clinical course indicative of a slight but clear progression after 3-years of follow-up. This contrasts the initial view on *SYNE1*-related ataxia as a relatively benign, slowly progressive condition, with important implications for clinic-genetic counselling.

## Declaration of Competing Interest

The authors declare that they have no known competing financial interests or personal relationships that could have appeared to influence the work reported in this paper.

## Footnotes

Appendix A Supplementary data to this article can be found online at https://doi.org/10.1016/j.prdoa.2023.100205.

## References

[1] Noreau A., Bourassa C.V., Szuto A., Levert A., Dobrzeniecka S., Gauthier J. (2013). SYNE1 mutations in autosomal recessive cerebellar ataxia. JAMA Neurol.

[2] Attali R., Warwar N., Israel A., Gurt I., McNally E., Puckelwartz M., Glick B., Nevo Y., Ben-Neriah Z., Melki J. (2009). Mutation of SYNE-1, encoding an essential component of the nuclear lamina, is responsible for autosomal recessive arthrogryposis. Hum Mol Genet..

[3] Gros-Louis F., Dupré N., Dion P., Fox M.A., Laurent S., Verreault S., Sanes J.R., Bouchard J.-P., Rouleau G.A. (2007). Mutations in SYNE1 lead to a newly discovered form of autosomal recessive cerebellar ataxia. Nat Genet.

[4] Synofzik M., Smets K., Mallart M. (2016). SYNE1 ataxia is a common recessive ataxia with major non-cerebellar features: a large scale muti-centre study. Brain.

[5] Mademan I., Harmuth F., Giordano I., Timmann D., Magri S., Deconinck T., Claaßen J., Jokisch D., Genc G., Di Bella D., Romito S., Schüle R., Züchner S., Taroni F., Klockgether T., Schöls L., De Jonghe P., Bauer P., Consortium EOA, Baets J., Synofzik M. (2016). Multisystemic SYNE1 ataxia: confirming the high frequency and extending the mutational and phenotypic spectrum. Brain.

[6] Bhatia K.P., Bain P., Bajaj N., Elble R.J., Hallett M., Louis E.D., Raethjen J., Stamelou M., Testa C.M., Deuschl G. (2018). Consensus Statement on the classification of tremors. from the task force on tremor of the International Parkinson and Movement Disorder Society: IPMDS Task Force on Tremor Consensus Statement. Mov Disord..

[7] Schmitz-Hubsch T., du Montcel S.T., Baliko L., Berciano J., Boesch S., Depondt C., Giunti P., Globas C., Infante J., Kang J.-S., Kremer B., Mariotti C., Melegh B., Pandolfo M., Rakowicz M., Ribai P., Rola R., Schols L., Szymanski S., van de Warrenburg B.P., Durr A., Klockgether T. (2006). Scale for the assessment and rating of ataxia: development of a new clinical scale. Neurology.

[8] Trouillas P., Takayanagi T., Hallett M., Currier R.D., Subramony S.H., Wessel K., Bryer A., Diener H.C., Massaquoi S., Gomez C.M., Coutinho P., Hamida M.B., Campanella G., Filla A., Schut L., Timann D., Honnorat J., Nighoghossian N., Manyam B. (1997). International Cooperative Ataxia Rating Scale for pharmacological assessment of the cerebellar syndrome: The Ataxia Neuropharmacology Committee of the World Federation of Neurology. J Neurol Sci.

